# Analytical approaches to account for muscle size when evaluating strength

**DOI:** 10.1007/s00421-026-06376-6

**Published:** 2026-08-17

**Authors:** Matthew A. Chatlaong, John P. Bentley, Kealey J. Wohlgemuth, Scott J. Dankel, Jeremy P. Loenneke, Matthew B. Jessee

**Affiliations:** 1 Exercise Science - School of Human Services, University of Cincinnati, Cincinnati, OH USA; 2 Department of Pharmacy Administration, School of Pharmacy, University of Mississippi, University, MS USA; 3 Department of Health and Exercise Science, Rowan University, Glassboro, NJ USA; 4 Kevser Ermin Applied Physiology Laboratory, Department of Health, Exercise Science, and Recreation Management, University of Mississippi, University, MS USA; 5 Applied Human Health and Physical Function Laboratory, Department of Health, Exercise Science, and Recreation Management, University of Mississippi, University, MS USA

**Keywords:** Resistance training, Hypertrophy, Specific tension, Muscle quality, Specific force, Normalization

## Abstract

Skeletal muscle strength is multifactorial. Although associated, skeletal muscle size and strength often change disproportionately following resistance training with different loading paradigms. Examining strength relative to muscle size has been used to evaluate the potential contribution of muscle growth or other factors (e.g., neural or intramuscular adaptations) that occur with strength changes. Moreover, as muscle size could explain differing strength between individuals, comparisons of strength per unit of muscle size are sometimes used to account for differences in strength related to size. Several analytical approaches can be used to account for or hold muscle size fixed when evaluating strength. Based on prior work in sports medicine and other fields, we explored the use of ratio normalization and multiple regression techniques. Data from NHANES (1999–2002) and a previous investigation from our group were used in analysis demonstrations. In synthesizing and applying recommendations from previous work, we highlight some nuances and complexities with ratio normalization that may not be readily apparent without testing assumptions.

## Introduction

Muscle size is strongly associated with muscle strength (Maden-Wilkinson et al. 2020). Notwithstanding, causal interpretation of the muscle size and strength relationship can be nuanced (Loenneke 2021; Folland and Balshaw 2021; Loenneke et al. 2019; Taber et al. 2019), and it is widely recognized that strength is multifactorial (Sale 1988; Moritani and deVries 1979). In any case, when investigating muscle strength, as an outcome or predictor of other variables, taking its relationship with muscle size into account is warranted. From a quantitative perspective, it follows that expressing strength relative to muscle size or mass^1^ (e.g., with ratios of strength/size) provides an index of theoretical interest. In the context of resistance training, these ratios, often referred to as specific tension (ST) are used to express muscle strength relative to muscle size at the whole muscle (Maden-Wilkinson et al. 2020; Erskine et al. 2010, 2011; Castro et al. 1995; Maughan et al. 1984) and single muscle fiber (Meijer et al. 2015; Frontera et al. 2003; Cristea et al. 2008) levels. Analogously, in aging research where declining muscle strength and mass is a focal point, muscle strength/size ratios have been termed “muscle quality” (MQ) (Barbat-Artigas et al. 2013; Fragala et al. 2015; McGregor et al. 2014), although not universally (Cawthon et al. 2022; Coronado-Zarco and de León 2023; de Lucena Alves et al. 2023).

As is common with ratios of variables in other fields, ST or MQ are typically used to (1) investigate proportionality (e.g., strength relative to size), or (2) adjust a variable of interest for the influence of the denominator (e.g., strength corrected for, accounting for, or normalized to size) (Smith 2005). Smith (2005) discusses these uses as conceptually distinct; the former focusing on the relationship between the two variables (i.e., one relative to the other) and the latter focusing on one variable (strength) while achieving statistical control of another (size).

Either way, using ratios of variables can provide valuable insight. As several authors in sports medicine (Atkinson and Batterham 2012; Atkinson et al. 2009; Nevill et al. 1992) and other fields (Curran-Everett 2013; Kronmal 1993; Packard and Boardman 1999; Atchley et al. 1976; Pearson 1896; Allison et al. 1995; Certo et al. 2020; Albrecht et al. 1993) have discussed, there are potentially underappreciated theoretical and quantitative complexities that can arise, however, when using them. The overarching goal of this investigation was to narratively review previous work outlining some of these complexities, with discussion of previously recommended solutions or alternatives. First, physiological interpretation of ST and MQ is briefly explored in the context of proportionality. Then, subsequent sections detail the primary focus of the review; accounting for the influence of muscle size when evaluating strength. Analysis demonstrations were conducted to explore ratio normalization and other recommended methods using two separate datasets. Lastly, some considerations for using ratios of variables in statistical analyses are discussed.

## Mechanistic perspectives: investigating strength relative to size

Studying ratios of muscle strength relative to size or mass has yielded valuable insight into the multifactorial nature of strength for decades (Ikai and Fukunaga 1968, 1970). ST and MQ have been considered markers of neuromuscular function, the interest being in the functional output (strength) of a given unit of muscle. This has been used to shed light on the relative contribution of muscle size amongst other plausible factors (e.g., neural or intramuscular determinants) to strength. For example, in the resistance training literature, cross sectional studies have demonstrated that ST can differ between muscle fibers from individuals with different training backgrounds. Meijer et al. (2015) found that muscle fibers from bodybuilders and power athletes produced ~ 33–50% higher force than recreationally active individuals, but that ST was ~ 41–63% lower in fibers from body builders than the other groups. This observation led the authors to hypothesize that excessive hypertrophy may have a detrimental effect on ST (Meijer et al. 2015). Moreover, in each group, a negative association was observed between ST and fiber cross-sectional area, indicating that greater fiber size was associated with lower relative strength (Meijer et al. 2015). Similar negative associations have been observed between MQ and muscle mass in aging research (Barbat-Artigas et al. 2012). These findings are open to several interesting physiological interpretations. On one hand, lower ST or MQ (potentially as a function of greater muscle size/mass) could indicate that additional muscle tissue may not be aiding in maximal force development. The composition or other properties of the greater muscle tissue may therefore warrant further study to understand the potential lack of, or diminished, functional contribution to maximal force development (Roberts et al. 2020; Meijer et al. 2015). On the other hand, the findings could be used to highlight dissociation between muscle size and strength across a range of contexts, raising questions about the contributory role of muscle size to muscle strength. With either interpretation, investigating proportionality of ST or MQ between or within groups raises questions about how muscle size and strength are linked.

Longitudinal observations from studies employing resistance training interventions also give rise to compelling physiological questions. With high-load resistance training, increases in strength (numerator) frequently outpace that of size (denominator), increasing ST or MQ (Erskine et al. 2011). Likewise, when training consists of one-repetition maximum efforts, dynamic muscle strength (e.g., one-repetition maximum) can increase substantially without appreciable muscle growth (Mattocks et al. 2017; Dankel et al. 2017, 2020; Jessee et al. 2021), which would also increase ST or MQ. When comparing results between high and low-load resistance training interventions, however, differing patterns of change in ST result (Reggiani and Schiaffino 2020). Both high-and low-load resistance training paradigms can drive comparable increases in muscle size (Currier et al. 2023; Lopez et al. 2021; Schoenfeld et al. 2017),^2^ although low-load training can result in small or even no changes dynamic strength (e.g., one-repetition maximum) (Jessee et al. 2018; Hammert et al. 2023; Jenkins et al. 2016). It follows then, that with low-load training, the denominator variable could increase with little or no increase in the numerator, possibly resulting in a *loss* of ST or MQ while high-load training could improve it. This could drive questions about the functional consequences of muscle growth following low-load training, but it could also be in contrast with theoretical perspectives that would argue training should increase muscle function in some manner regardless of the load used. Adding to these divergent responses, in the case of detraining, strength (numerator) can be maintained despite decreasing muscle size (denominator), leading to an *increase* in MQ or ST (Bickel et al. 2011). Accordingly, depending on the case, opposing stimuli (e.g., high-load training and detraining) could result in a change in the ratio of two variables in the same direction due to differing effects on the numerator in one and denominator in the other. For those with the theoretical perspective that training should increase muscle function and detraining should decrease it, such results might appear contradictory.

Since two variables are involved in a single metric with a ratio, some have argued that a range of possible numerator and denominator combinations complicates interpretation of the ratio score (Allison et al. 1995). It is interesting to note, however, that by using ST and MQ to assess proportionality (interpreting the proportion as an indicator of muscle quality/function), the potentially conflicting interpretations outlined above could be considered informative in their own right. For example, depending on perspective, it could be suggested that the quality/function of a given amount of muscle tissue could increase throughout training, and possibly further by reducing training or detraining (e.g., due to diminishing or detrimental effects of muscle growth on maximal strength requiring further investigation). It could also be argued that contradictions highlight a different issue – that changing muscle size or mass does not inherently *have to* be proportional with differences in strength (Clark and Manini 2008; Loenneke 2021). In this latter perspective, independent changes in numerator and denominator might could suggest that muscle size and strength may not have a deterministic relationship as is implicit in interpreting the ratio as quality/function. Instead, it might be argued that making muscle size and strength dependent on each other is theoretically inconsistent. Either way, the assessment of strength relative to muscle size highlights several unresolved questions about the relationship between muscle size and strength.

## Normalizing or accounting for size when investigating strength

Often, the objective of forming ratios of variables is to normalize or scale the numerator (strength) for the denominator (size), or in other words, to account for or remove the effects of the denominator (Albrecht et al. 1993; Allison et al. 1995; Atkinson and Batterham 2012; Curran-Everett 2013; Kronmal 1993; Packard and Boardman 1988, 1999). As suggested by Smith (2005), the theoretical focus is on studying a single trait in this case, i.e., strength, with statistical control of differences related to size. As most physiological variables can vary with size (e.g., muscle blood flow and oxygen consumption), adjusting them to remove the effects of size is important for understanding other causative factors (Packard and Boardman 1999).

**Fig. 1:**
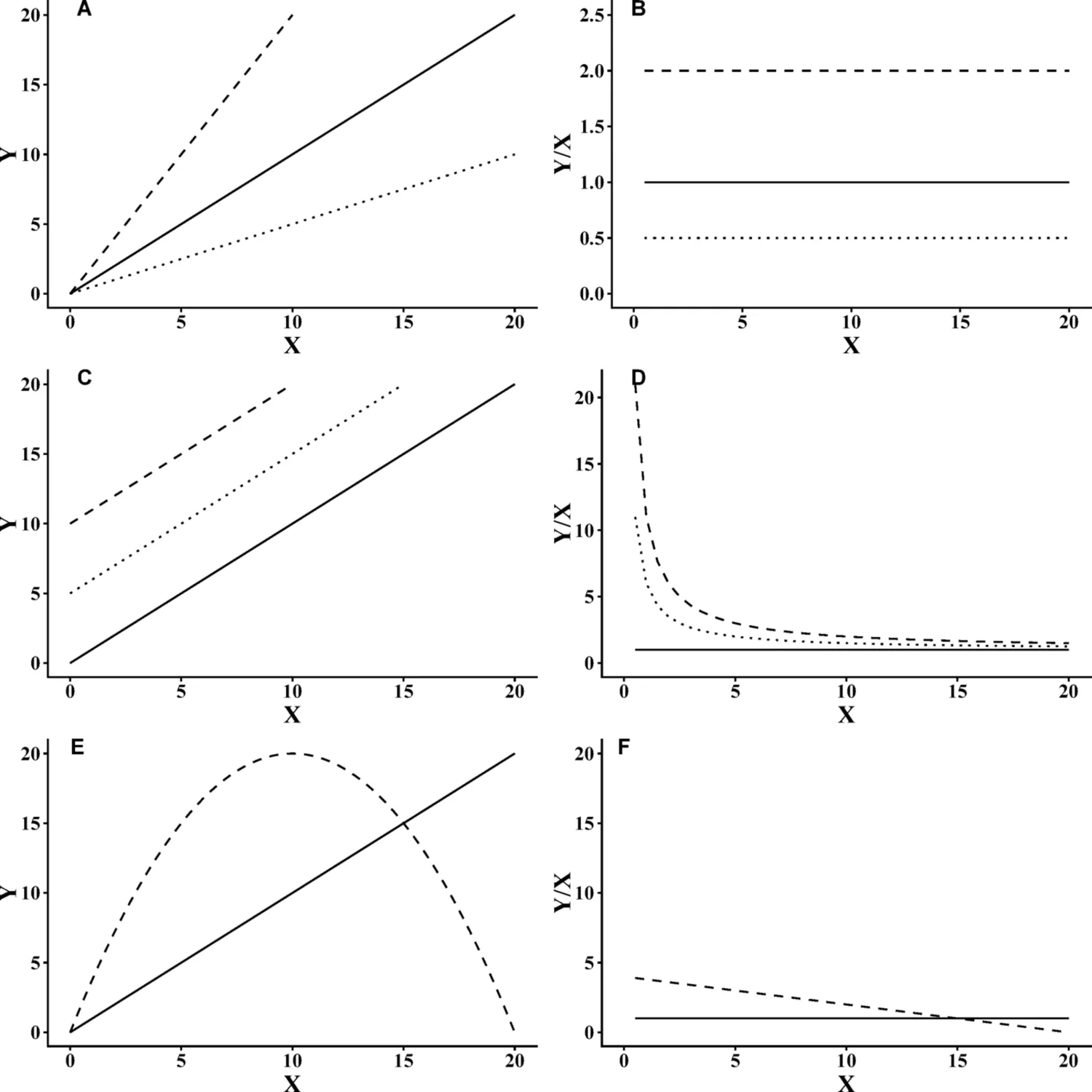
Relationships for hypothetical data of numerator (*Y*) and denominator (*X*) variables. Panels on the left show the numerator (*Y*) plotted against the denominator and panels on the right show corresponding ratios (*Y/X*) plotted against the denominator (*X*). **A** Three different linear *X*-*Y* relationships with different slopes that pass through the origin. **B** Corresponding ratio computed from *X* and *Y* variables in A, plotted against *X*. Flat lines suggest no systematic relationship between *Y*/*X* and *X*, i.e., even scaling. **C** Three different linear *X*-*Y* relationships with differing y-intercept values but the same slope. **D** Corresponding ratio computed from *X* and *Y* variables in C, plotted against *X*. Note the systematic relationships between *Y*/*X* and *X* when lines in C do not pass through the origin, i.e., uneven scaling. **E** Curvilinear *X*-*Y* relationship compared with a linear one that passes through the origin. **F** Corresponding ratio computed from *X* and *Y* variables in E, where there is a systematic relationship between *Y*/*X* and *X* for the curvilinear data

It is not always recognized, however, that unintended consequences could arise when forming a ratio of variables to achieve control of the denominator (Allison et al. 1995; Albrecht et al. 1993; Atkinson and Batterham 2012; Curran-Everett 2013; Packard and Boardman 1999). Importantly, in forming a ratio with numerator *Y* and denominator *X* to scale or normalize *Y* for *X*, it is implicit that *Y* and *X* have a linear “isometric” relationship (Packard and Boardman 1988; Albrecht et al. 1993). Packard and Boardman (1988, 1999) describe an isometric relationship as a proportional change in the variables, where a plot of *Y* and *X* generates a line of best fit that is straight and passes through the origin of the graph (Fig. 1) (i.e., it has a y-intercept of 0). Albrecht et al. (1993) highlight that the underlying structure of the data is assumed to be best described by this model,^3^ represented by Eq. 1:

1 $$\:Y=\:b\cdot\:X$$

Where *Y*, the numerator or variable of interest (e.g., strength), is linearly related to *X*, the denominator (e.g., size) by a straight line that passes through the origin with a slope coefficient *b*.

If the condition of isometry is met, then the ratio of *Y/X* will scale evenly across values of *X* (e.g., will not be correlated with *X*), but if unmet, the ratio and its denominator may be related (Fig. 1) (Allison et al. 1995; Atkinson and Batterham 2012; Curran-Everett 2013; Packard and Boardman 1999; Albrecht et al. 1993). Curran-Everett (2013) has provided R code for example demonstrations of this effect. Issues could arise if ST or MQ were being compared between two groups to answer the question of which is stronger after normalizing for or removing the influence of size (Table 1). If ST or MQ were negatively correlated with size (Barbat-Artigas et al. 2012; Meijer et al. 2015), a group with smaller muscles could have higher ST or MQ than one with larger muscles due to uncontrolled influence of size on the ratio scores (Allison et al. 1995; Curran-Everett 2013; Kronmal 1993; Packard and Boardman 1999). Smith (2005) suggests that interpretation of relationships between a ratio and its denominator is nuanced and likely depends on whether the intent in forming a ratio is to control for the effect of the denominator, or if it is to investigate implications of proportionality. Smith (2005) highlights that in the latter case, a ratio may represent a unique physiological construct with distinct implications, and accordingly whether any difference in ratio scores due to differences in the denominator may or may not be of interest. Though if the ratio score is intended to represent the numerator controlled for the denominator, relationships between the ratio and its denominator could lead to spurious conclusions about the trait of interest (Smith 2005; Albrecht et al. 1993; Allison et al. 1995; Curran-Everett 2013; Kronmal 1993; Lolli et al. 2017a; Packard and Boardman 1999), i.e., strength. Accordingly, if a ratio of variables is used to control for or normalize the numerator variable for the denominator, it is important to assess their underlying relationship.

### Assessing isometry with muscle size and strength outcomes

To assess whether the linear relationship between *X* and *Y* is described by a line of best fit passing through the origin, i.e., has a y-intercept of 0 (Allison et al. 1995; Packard and Boardman 1999), a regression line must be fit to their bivariate data (Curran-Everett 2013; Warton et al. 2006; Albrecht et al. 1993). Note that the y-intercept must be allowed to vary and estimated, i.e., the intercept cannot be omitted from the regression model to force the line through the origin. To determine the line of best fit when evaluating isometry, standardized major axis regression (SMA) (also known as reduced major axis regression or model II regression) is sometimes recommended instead of the ordinary least squares (OLS) approach (Warton et al. 2006; Smith 2009). The regression procedure influences line fit, and thus the estimate of the y-intercept. Briefly, the SMA approach fits the line by minimizing deviations in both the *X* and *Y* directions, versus OLS minimizing deviations in the *Y* direction (Warton et al. 2006). For isometry testing, the goal is to summarize the pattern of scaling between the two variables, assessing whether they change proportionally, which differs from using regression to estimate values of one variable given fixed values of the other (Warton et al. 2006). For a thorough review comparing different line-fitting methods, as well as the assumptions and applications of each, see Warton et al. (2006) and Smith (2009). Various R software packages are available for implementing SMA regression, including the smatr() package (Warton et al. 2012) and the lmodel2() package as demonstrated by Curran-Everett (2013). The smatr() package was used for the analysis demonstrations herein.

Recommended approaches are demonstrated in the following sections using two different sets of muscle size and strength variables. First, to assess the relationship between muscle size and strength at a population level, we utilized leg muscle mass and strength measures from 2,848 participants from the 1999–2002 exam cycles of the National Health and Nutrition Examination Survey (NHANES). Second, to explore isometry when working within smaller sample datasets with other size and strength variables (i.e., should researchers be interested in making a ratio from different variables), we utilized previously collected data from our laboratory.

### Example 1: NHANES

In NHANES exam cycles from 1999 to 2002, muscle size and strength outcomes were used to generate ratios of variables in a total of *n* = 1409 female and *n* = 1439 male participants. Muscle strength was quantified as unilateral isokinetic (60 degrees/sec) knee extensor torque measured via dynamometer, and leg lean mass (excluding bone content) was quantified via dual x-ray absorptiometry (DXA). Both measures were taken in the right leg. Given the nature of this demonstration analysis, the complex sampling design features (i.e., weights, clusters, strata) of the NHANES were not incorporated.

**Fig. 2:**
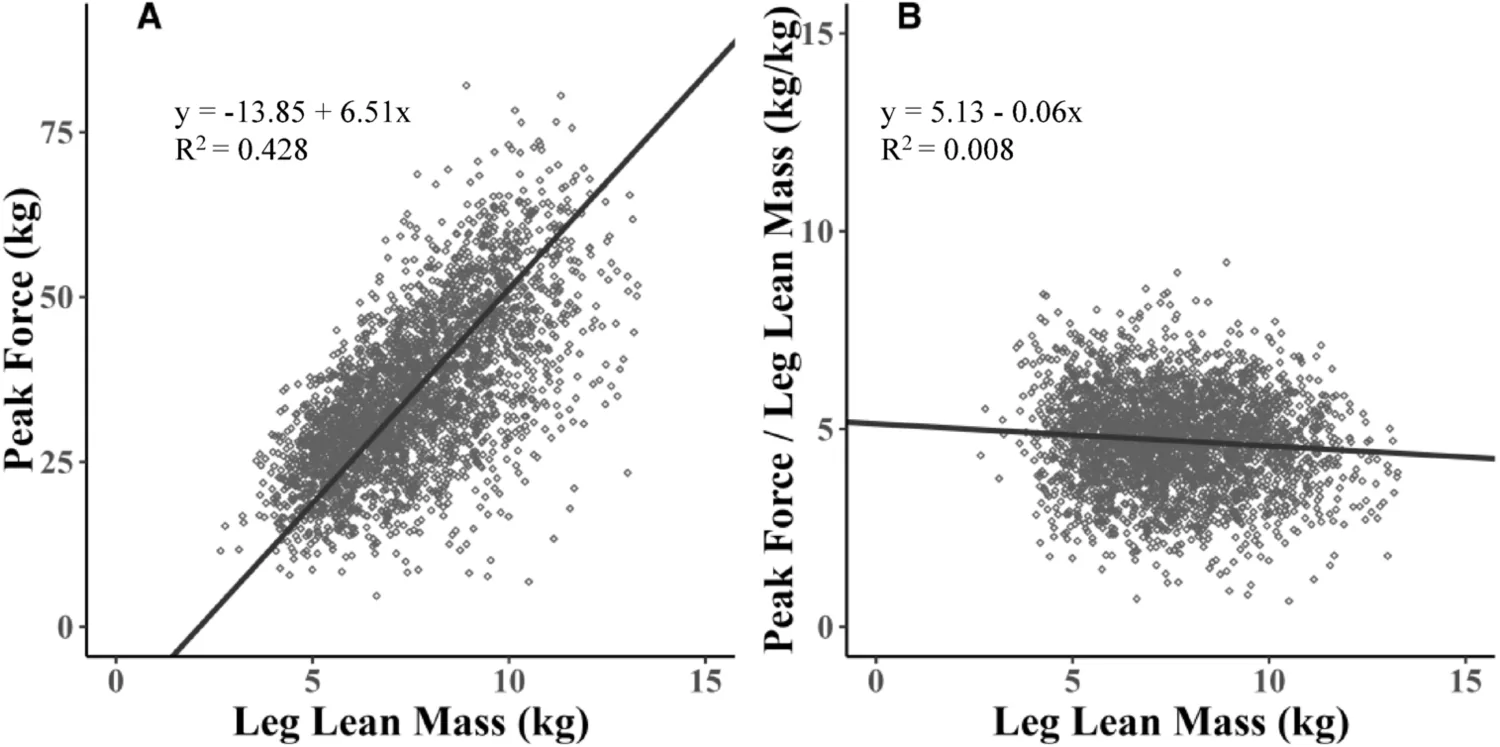
**A** SMA fit of peak isokinetic force (kg) regressed on leg lean mass (kg). **B **OLS fit of the ratio of peak isokinetic force/leg lean mass (kg) regressed on the denominator leg lean mass (kg)

To begin, the numerator variable, peak isokinetic force (kg) (*Y*), was regressed on the denominator variable, leg lean mass (kg) (*X*), using SMA (Fig. 2A) (Warton et al. 2006, 2012). For the condition of isometry to be met, the regression coefficient for the y-intercept should be 0, though was estimated at -13.85 kg (95% CI: -15.27 to -12.43, *p* < 0.001, Fig. 2A). If a ratio, *Y/X*, was formed, it may be systematically related to the denominator (Allison et al. 1995). To evaluate this, the ratio of peak force (*Y*) to leg lean mass (*X*) was plotted against leg lean mass (*X*) to visualize any potential relationships (Fig. 2B). Of note, fitting a straight line to the plot in Fig. 2B may not characterize the relationship between the ratio and denominator. This is shown, for example, in some possible curvilinear relationships between ratios and denominator variables in Fig. 1D.

That said, we tested for a linear relationship between *Y/X* and *X* for simplicity, i.e., a linear model was fitted where *Y/X* was regressed on *X* (Fig. 2B). OLS regression was used, as the goal was not to assess isometry between the ratio and denominator, but rather systematic differences in the ratio given values of *X* (Albrecht et al. 1993). In this case, the non-zero slope coefficient of -0.06 units (95% CI: -0.08 to -0.03, *p* < 0.001) suggests a linear relationship that could warrant cautious interpretation of the ratio (Fig. 2B). The slope coefficient suggests that scores for ratio are systematically *lower* for those with *greater* leg lean mass (Fig. 2B). For example, if the expected MQ or ST were compared between those with 5 kg of leg lean mass and those with 10 kg, there would be an expected difference of 0.30 kg/kg between them, imposed by a lack of isometry. Of course, this effect of 0.30 kg/kg could be considered practically or theoretically inconsequential, but effect sizes could vary with different outcomes, samples, etc., and if severe, could affect conclusions when comparing people with high and low leg lean mass if it assumed that size is controlled for.

### Example 2: sample data from our laboratory

Often research studies are conducted with smaller samples and could involve a wide variety of muscle size and strength measures. This means that results of the analysis demonstration using the NHANES data could differ substantially from results in other studies. To explore this, we used previously collected pre-training data from a resistance training study conducted by our group (*n* = 94 female, *n* = 57 male) (Dankel et al. 2020). Strength was quantified as dynamic one-repetition maximum (1RM) for the bicep curl in the dominant arm, and muscle size was quantified using B Mode ultrasound to measure the thickness of the muscle at the midpoint of the acromion and lateral epicondyle (Dankel et al. 2020).

**Fig. 3:**
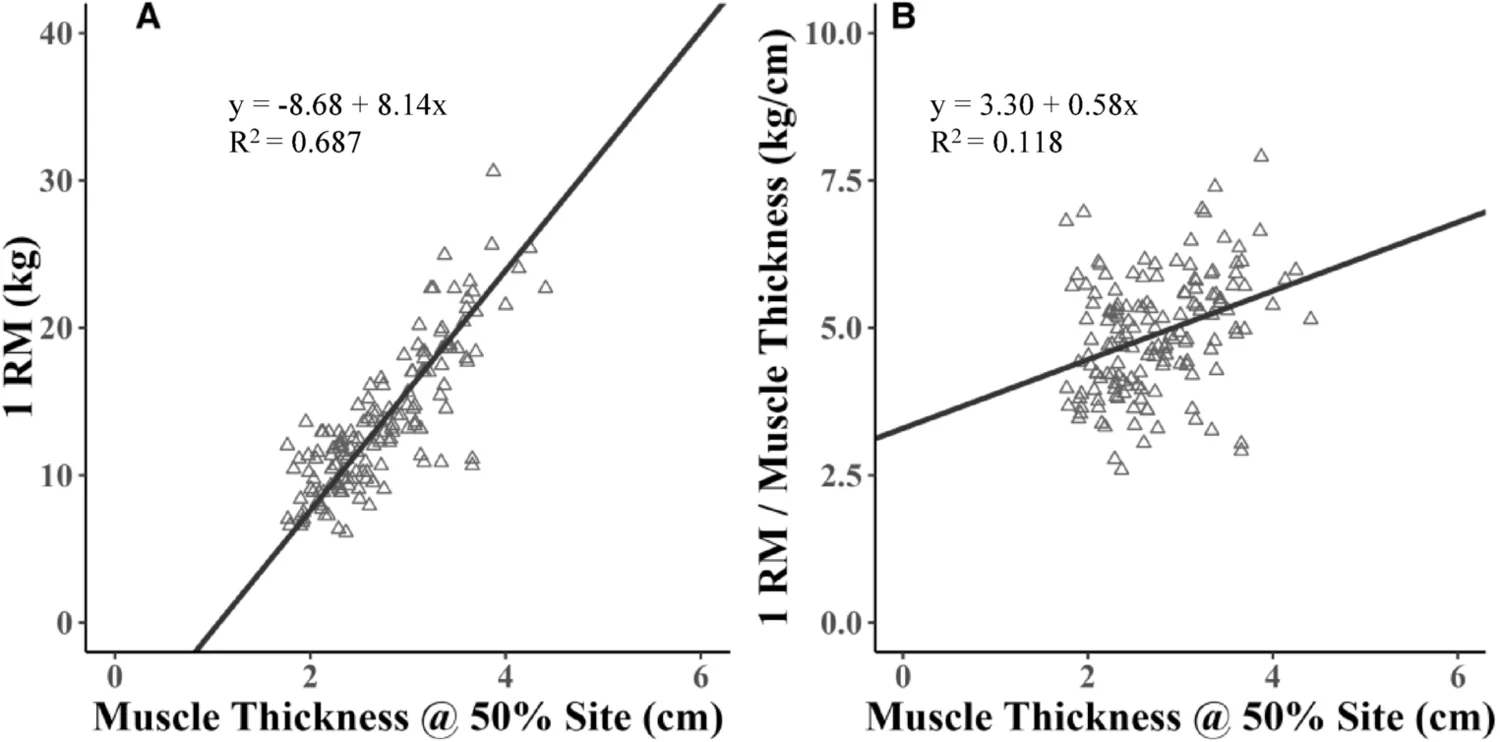
**A** SMA fit of one-repetition maximum (1RM) strength (kg) regressed on muscle thickness (cm). **B** OLS fit of the ratio of 1RM/muscle thickness (kg/cm) regressed on the denominator muscle thickness (kg)

Similarly, the numerator, 1RM strength, was regressed on the denominator, ultrasound muscle thickness, using SMA (Fig. 3A). The non-zero y-intercept of this line (-8.68 kg, 95% CI: -10.74 to -6.62, *p* < 0.001) indicates a lack of isometry. Similar to the NHANES example, the ratio of 1RM/muscle thickness (kg/cm) was plotted against the denominator and the linear relationship between *Y/X* and *X* was assessed using OLS regression (Fig. 3B). The slope coefficient in Fig. 3B indicates that on average, ratio scores are 0.58 kg/cm higher (95% CI: 0.32 to 0.84, *p* < 0.001) for each additional cm of muscle thickness. Interestingly, the effect of uneven scaling occurs in the opposite direction compared with the NHANES example (i.e., in that example higher muscle mass was associated with *lower* ratio scores). This may be due to differing measures or participant characteristics between the datasets. Nonetheless, it indicates that results may vary across studies.

### Isometry testing: decision-making with hypothesis tests

Both isometry testing analysis demonstrations indicated that y-intercepts were significantly different from 0, and that uneven scaling of the ratio could result. However, for several reasons, Allison et al. (1995) suggest the hypothesis test of the intercept might be a “questionable heuristic” to decide about proceeding with a ratio. For example, in smaller samples, is statistical power sufficient to reject the null hypothesis that the y-intercept = 0 (Allison et al. 1995)? Moreover, Allison et al. (1995) point out that the intercept, a continuous variable, cannot equal *exactly* zero. Do small deviations from a 0 intercept warrant concerns with uneven scaling? Notably, the ratio could still be systematically related to, or significantly correlated with, its denominator if the intercept is not “statistically different” from 0 (Allison et al. 1995). There clear criteria for determining whether a y-intercept is too large. Determining acceptable limits for its magnitude could be theoretically/practically challenging. Thus, an additional precaution, as discussed by Allison et al. (1995), could be to use a liberal alpha level (e.g., 0.20) when testing for y-intercept = 0 and more conservative alpha levels (e.g., 0.01 instead of 0.05) for substantive hypothesis tests involving the ratio.

Although debatable, it could be argued that statistical inference (i.e., hypothesis testing and p values) for isometry tests might be unnecessary if the concern is scaling *within* a sample versus using the sample to infer isometry in the parent population. In other words, hypothesis testing may help determine if the “simple random sample” at hand has a lack of isometry by chance (i.e., from sampling variation) or not. Consequently, one could also suggest that subjective evaluation of y-intercept and the relationship between the ratio and its denominator may be used to decide whether there is a pattern that is concerning. Overall, inherent subjectivity in the process of establishing isometry can create difficulty in proving adequate control of the denominator variable (Packard and Boardman 1999).

### Ratios adjusted for the y-intercept

Given that a non-zero y-intercept indicates a lack of isometry, adjusting, offsetting, or centering the *Y* variable on the non-zero y-intercept value presents an intriguing solution. The y-intercept estimate, obtained from regressing *Y* on *X* before forming the ratio, would be subtracted from each score on the original *Y* variable (Corruccini 1977; Allison et al. 1995; Albrecht et al. 1993) to form a new intercept-adjusted *Y* variable, *Y-a*, where a is the estimate of the y-intercept. *Y-a* would be used instead of *Y* to generate the ratio, (*Y-a*)/*X*. Note that this procedure forces the line of best fit between *Y-a* and *X* through the origin, but it is not, however, the same as estimating a regression equation between *Y* and *X* where the intercept estimate is omitted. Some have suggested that this approach can be effective (Corruccini 1977; Albrecht et al. 1993), while others have demonstrated remaining relationships between the ratio and denominator (Atchley 1978). To explore this approach, we applied it to our previously collected sample of muscle size and strength data (Example 2).

**Fig. 4:**
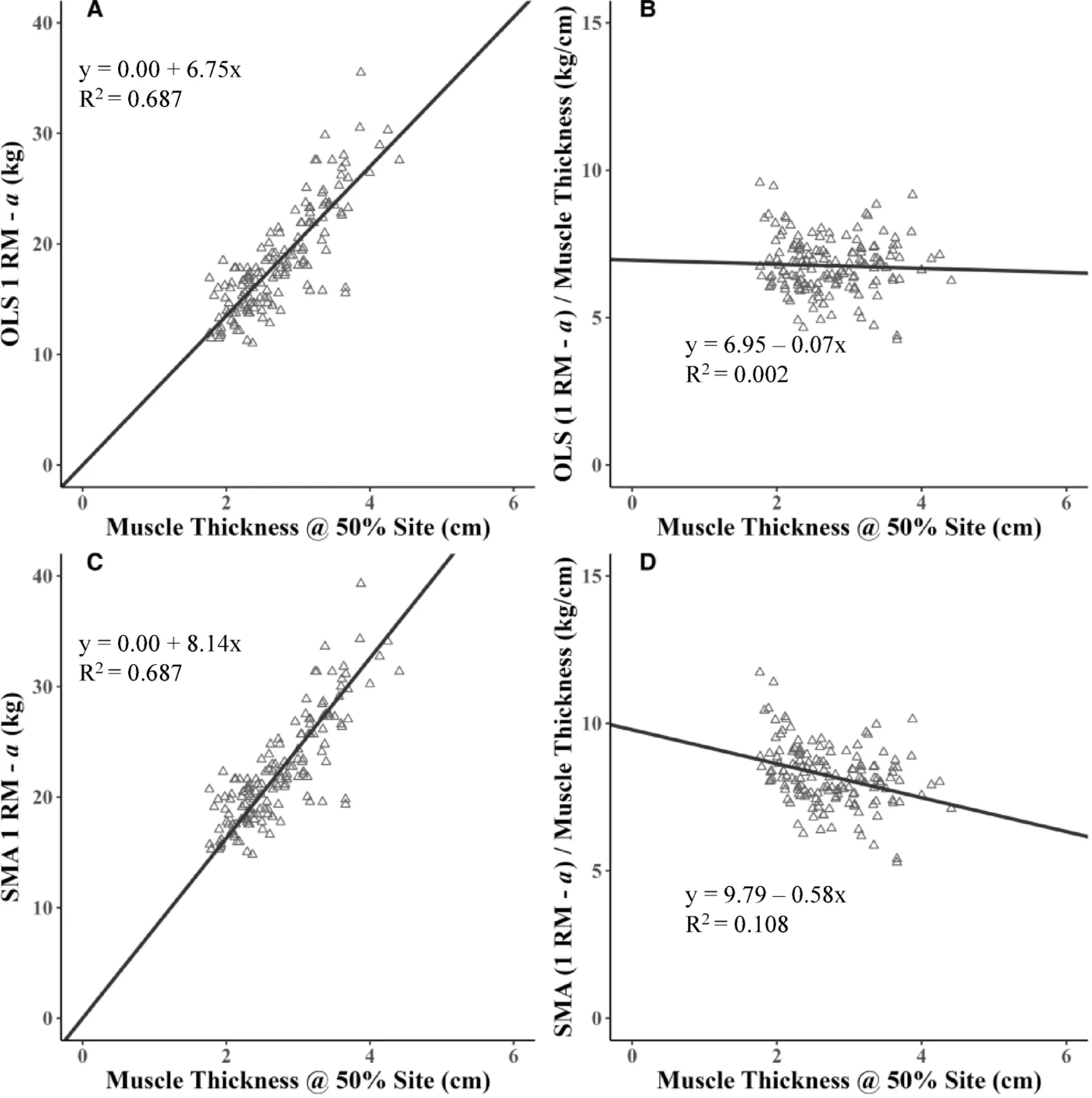
**A** OLS fit of y-intercept adjusted 1RM strength (kg) regressed on muscle thickness (cm). The y-intercept (*a*) used for adjustment (1RM - *a*) was estimated using OLS. **B** OLS fit of the ratio of y-intercept adjusted 1RM strength/muscle thickness (kg/cm) regressed on muscle thickness (kg). **C** SMA fit of y-intercept adjusted 1RM strength (kg) regressed on muscle thickness (cm). The y-intercept (*a*) used for adjustment in this case was estimated using SMA. **D** SMA fit of the ratio of y-intercept adjusted 1RM strength/muscle thickness (kg/cm) regressed on muscle thickness (kg). Notice that the scaling issue remains in this plot, since the ratio was formed using intercept adjustment from SMA estimation *(*Albrecht et al. 1993)

First, the y-intercept was estimated. Importantly, when performing intercept-adjustment to form *Y-a*, the y-intercept estimate should be from OLS regression of *Y* on *X* (not SMA) or correlations between the intercept-adjusted ratio (*Y-a*)/*X* and the denominator *X* could remain (Albrecht et al. 1993) Figs. 4A and B show results when following the recommendations discussed by Albrecht et al. (1993). 1RM strength regressed on muscle thickness using OLS yielded an estimated y-intercept of -4.89 kg (*p* < 0.001). Note that this differs from the y-intercept estimate of -8.68 kg obtained from using SMA previously (Fig. 3A). The OLS y-intercept estimate of -4.89 was subtracted from each score on the original 1RM variable to yield a new adjusted numerator variable (y-axis in Fig. 4A). Next, the adjusted numerator variable was regressed on the denominator using OLS, verifying that the OLS line of best fit passed through the origin (Fig. 4A). The adjusted 1RM variable (*Y-a*) was divided by muscle thickness (*X*) to form the ratio, (*Y-a*)/*X*, and plotted against the denominator *X* (Fig. 4B). The systematic relationship previously observed between *Y/X* and *X* (Fig. 3B) was largely eliminated. The ratio (*Y-a*)/*X* was estimated to decrease by 0.07 kg (95% CI: -0.33 to 0.19) per cm increase in muscle thickness, which was not statistically significant (*p* = 0.601). Note that the slope in Fig. 4B appears nearly flat. Interestingly, Figs. 4C and D show results from forming (*Y-a*)/*X* by using the SMA estimated y-intercept (-8.68 kg) instead of the OLS estimate (-4.89 kg). As suggested by Albrecht et al. (1993), there is a relationship between the ratio and the denominator after intercept adjustment when the SMA estimate is used.

Forming a ratio from (*Y-a*)/*X* appeared to alleviate uneven scaling observed with the “unadjusted” ratio of *Y*/*X* (Fig. 3B). Accordingly, if (*Y-a*)/*X* was being used to compare strength between two groups after normalizing for or removing the influence of size, (*Y-a*)/*X* may better accomplish the adjustment than *Y*/*X* (Table 1). That said, for several reasons some would still caution against the use of intercept-adjusted ratios (Allison et al. 1995; Albrecht et al. 1993; Atchley 1978). Notably, calculating a ratio of variables can impose systematic effects on the error distribution of the ratio with respect to its denominator (Allison et al. 1995; Albrecht et al. 1993). For example, if errors are homoscedastic when *Y* is regressed on *X*, then the ratio will likely be heteroscedastic relative to *X* or any variables that are correlated with *X* (Allison et al. 1995; Albrecht et al. 1993). Moreover, the ratio of two normally-distributed variables cannot be normally distributed (Albrecht et al. 1993; Allison et al. 1995; Atchley et al. 1976). The ratio of two independent normal variables is a Cauchy random variable, while the ratio of any two normal variables has a Cauchy-like distribution (Marsaglia 2006), which can present further analytical challenges, e.g., with statistical inference. Accordingly, ratios of variables may not readily meet some assumptions of regression analyses and parametric tests without further transformation (Allison et al. 1995; Smith 2005). Lastly, some have highlighted that the reliability of a ratio can be lower than that of its individual numerator and denominator variables (Allison et al. 1995), and that dispersion of denominator values can affect results (Certo et al. 2020). Thus, ratios formed from components that have limited reliability (i.e., have measurement error) will have attenuated reliabilities (i.e., propagation of error). Such issues would be expected even with intercept-adjustment, as subtracting a constant from each score on the numerator variable leaves the distributional properties of the variables unchanged (Atchley 1978).

### Allometric scaling and ratios adjusted for scaling exponents

A linear model has been assumed as the underlying process by which *X* and *Y* scale (Albrecht et al. 1993). However, theory could also suggest that the alternative to isometry would be an allometric relationship, commonly described by a power law model of a non-linear relationship (Eq. 2) (Albrecht et al. 1993; Nevill and Holder 1995; Nevill et al. 1992; Batterham et al. 1997):

2 $$\:Y=\:b\cdot\:{X}^{k}$$

where *Y*, the numerator or variable of interest (e.g., strength), is curvilinearly related to *X*, the denominator (e.g., size) by a power function defined by constants *b* and *k*. The constant *k* is an exponential term that is has been referred to as the allometric scaling exponent (Atkinson and Batterham 2013; Lolli et al. 2017b) or coefficient of allometry (Albrecht et al. 1993). As opposed to *Y* scaling in direct proportion with *X* as is the case in the simple linear model (Eq. 1), the exponent *k* describes the relationship between proportional changes in *Y* and proportional changes in *X* (Cohen et al. 2003). However, if *k* = 1, *Y* scales in direct proportion with *X*, and the model would be represented by a straight line passing through the origin, i.e., isometry (Albrecht et al. 1993; Atkinson and Batterham 2012). Thus, assessing whether *k* = 1 can inform whether the condition of isometry is met and may provide insight into potential allometric relationships that could theoretically align with the underlying scaling process of *X* and *Y*. Moreover, the allometric scaling exponent can be used to derive “allometrically adjusted ratios” (Albrecht et al. 1993) or “power function ratios” (Nevill et al. 1992), Y/*X*^*k*^, to normalize or remove the effects of size from the variable of interest. As with exploring ratios adjusted for the y-intercept (see section titled “*Ratios adjusted for the y-intercept*”) we examined allometric scaling and derived an allometrically adjusted ratio *Y*/*X*^*k*^ using the previously collected data from our laboratory.

First, *b* and *k* (Eq. 2) were estimated by fitting a log-log linear regression model of *Y* on *X* using OLS (Nevill et al. 1992). Although the power law model can quantify non-linearity in the scaling of *X* and *Y*, logarithmic transformation of *X* and *Y* linearizes the power function relationship in Eq. 2, meaning that coefficients *b* and *k* can be estimated via linear regression of log(*Y*) on log(*X*) (Batterham et al. 1997; Albrecht et al. 1993; Cohen et al. 2003; Nevill et al. 1992) as in Eq. 3:

3 $$\:\mathrm{log}\left(Y\right)=\mathrm{log}\left(b\right)+k\cdot\:\mathrm{l}\mathrm{o}\mathrm{g}\left(X\right)$$

where the estimate of *b* for Eq. 2 is obtained by exponentiating or taking the antilog of y-intercept coefficient from linear regression in Eq. 3, and the estimate of *k* for Eq. 2 is the estimated slope coefficient from linear regression in Eq. 3 (Cohen et al. 2003). It is worth highlighting that traditional allometric models assume that errors are multiplicative as opposed to additive on the original scale of the variables (errors are assumed to be additive on the log scale), which may actually align with many physiological processes that appear heteroscedastic across the range of size (Nevill et al. 1992; Nevill and Holder 1995). A multiplicative error term may also provide flexibility and intuitive interpretation when questions involve multiplicative relationships such as percent differences or changes (Nevill et al. 1992; Atkinson and Batterham 2013). While OLS regression with log-transformed variables is widely used to estimate *b* and *k*, non-linear regression techniques with variables on their original scale can also be used (Packard 2014, 2017b). Non-linear regression has been advocated for more flexible error assumptions and avoidance of possible bias introduced by transformation (Packard 2014, 2017b). While we utilize OLS regression following log transformations to demonstrate deriving an allometrically adjusted ratio *Y*/*X*^*k*^ (Albrecht et al. 1993),^4^ it should be highlighted that several allometric models could be fitted directly to untransformed data with non-linear regression procedures (Lolli et al. 2017a; Packard 2017a).

**Fig. 5:**
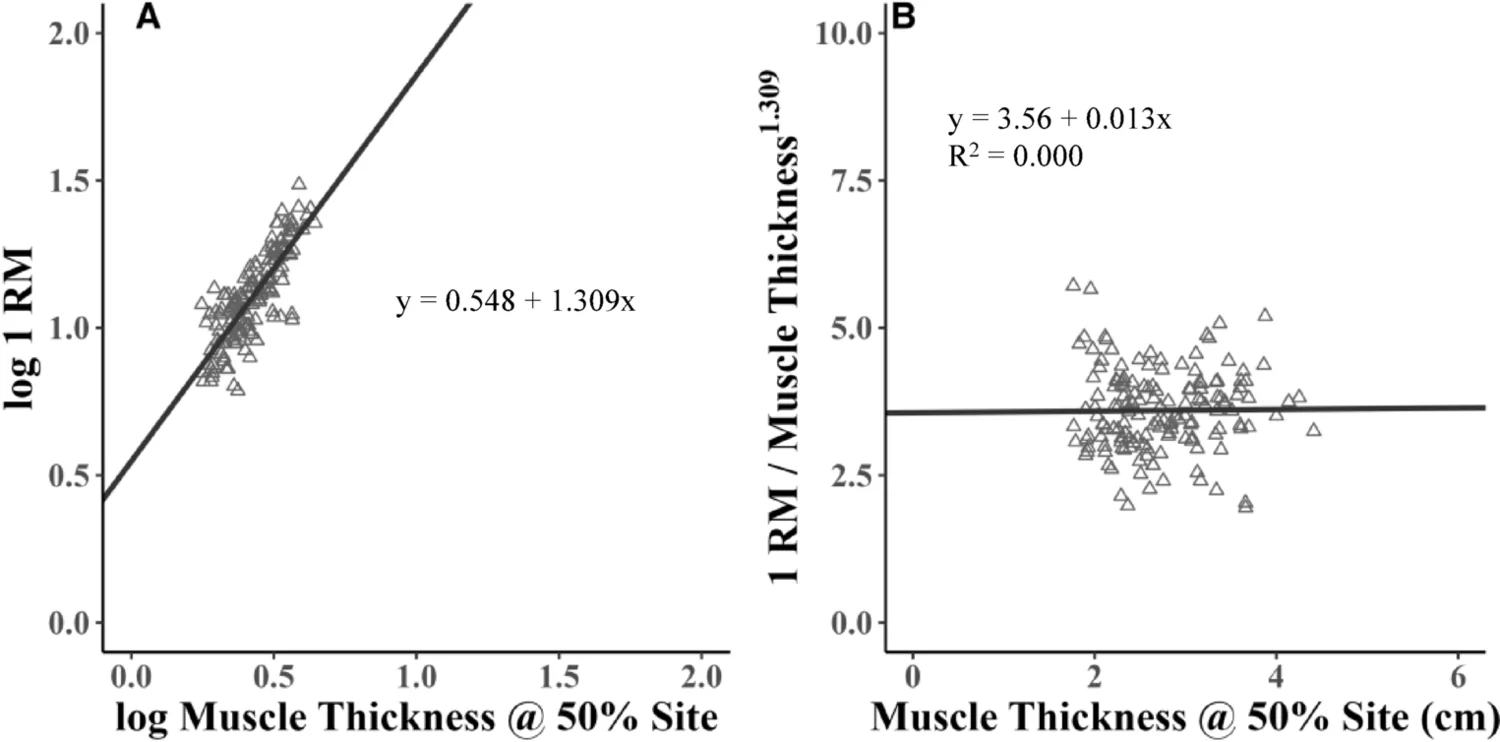
**A** OLS fit of log transformed 1RM strength regressed on log transformed muscle thickness. **B** OLS fit of the ratio of 1RM strength/muscle thickness exponentiated to the *k* power

Figure 5A shows the OLS regression of log(*Y*) on log(*X*) as denoted by Eq. 3. The slope coefficient of 1.309 (95% CI: 1.157 to 1.462) was significantly greater than 1 (*p* < 0.001),^5^^,^^6^ indicating that 1RM is expected to increase at a proportionally greater rate than muscle size. An interpretation could be that as muscle thickness increases by 1%, 1RM is expected to be ~ 1.3% greater, where under the condition of isometry, the expected difference in 1RM would remain 1%. Accordingly, the estimated value of *k* = 1.309 was used to calculate an allometrically adjusted ratio of *Y*/*X*^*k*^, as shown on the y-axis of Fig. 5B. Using OLS, the allometrically adjusted ratio was regressed on muscle size (Fig. 5B), yielding a slope coefficient of 0.013 (95% CI: -0.180 to 0.205, *p* = 0.895) which indicates that the scores for the allometrically adjusted ratio *Y*/*X*^*k*^ were not correlated with *X*.

As with deriving a ratio adjusted for the y-intercept (see section titled “*Ratios adjusted for the y-intercept*”), forming a ratio *Y*/*X*^*k*^ appeared to alleviate relationships between scores for the ratio *Y*/*X* and *X* that were observed in Fig. 3B. Accordingly, if used to compare two groups on strength after normalizing for or removing the influence of size (Table 1), even scaling may be better accomplished by *Y*/*X*^*k*^ or (*Y-a*)/*X* than with *Y*/*X*. However, as with ratios adjusted for the y-intercept, some would still express caution in using allometrically adjusted ratios for statistical control given size-related distributional effects on the ratio scores that can violate statistical assumptions and limit interpretability (Albrecht et al. 1993).

**Table 1:** Examples of group comparisons in strength when accounting for size with ratios of variables and regression-based approaches. General (non-exhaustive) considerations are provided for each approach

	Example group comparison	Potential estimand	Considerations
Simple ratio	Y/X ~ G ^1^	Group (*G*) difference in strength (*Y*) normalized for or accounting for size (*X*)	Assumes linear isometric relationship between *X* and *Y*. Magnitude may be affected by size. Consider assumptions for regression analysis.
Ratio adjusted for the y-intercept	(*Y*–*a*) /*X*~ *G *^2^	Group (*G*) difference in strength (*Y*) normalized for or accounting for size (*X*)	Assumes linear relationship between *X* and *Y*. Subtracting the y-intercept (*a*) from each *Y* may reduce or eliminate size effects on ratio scores, though ratio scores can contain size related distributional effects.^3^ Consider assumptions for regression analysis.
Allometrically adjusted ratio	*Y*/*X*^*k*^ ~ G or (*Y*-*a*)/*X*^*k*^ ~ *G* ^4^	Group (*G*) difference in strength (*Y*) normalized for or accounting for size (*X*)	Assumes non-linear relationship between *X* and *Y.* Consider possible group differences in allometric scaling exponents. Magnitude may be free of size effects, though ratio scores may have size related distributional effects.^3^ Consider assumptions for regression analysis.
Multiple regression/ANCOVA	*Y* ~ *X* + *G*	Group (*G*) difference in strength (*Y*) after accounting for size (*X*), equating on size, or considering size at a fixed value	Assumes linear relationship between *X* and *Y.* Consider assumptions of ANCOVA. May not be appropriate if groups do not overlap in size or size effects are group dependent. Consider assumptions for regression analysis.
Multiple regression/ANCOVA with log transformations	log(*Y*) ~ log(*X*) + *G *^5^	Multiplicative or proportional (*G*) difference in strength (*Y*) after accounting for allometric relationship with size (*X*)	Assumes log-log relationship between *X* and *Y.* Consider assumptions of ANCOVA. May not be appropriate if groups do not overlap in size or size exponents are group dependent. Consider assumptions for regression analysis.

^1^Note that *Y/X* ~ *G* refers to the *Y/X* ratio being predicted by a grouping variable, *G*, to determine differences between groups.

^2^The y-intercept (a) is estimated from ordinary least squares regression of *Y* on *X*. See section titled “*Ratios adjusted for the y-intercept”.*

^3^As noted by Albrecht et al. (1993), size-related distributional effects could complicate interpretation of what adjusted ratios represent.

^4^The allometric exponent *k* can be estimated from ordinary least squares regression of log(*Y*) on log(*X*). The allometric exponent *k*, and y-intercept *a* in adjusted ratio (*Y*-a)/*X*^*k*^, described by Albrecht et al. (1993) as a “Fully adjusted ratio”, can be estimated using non-linear regression procedures.

^5^Nevill et al. (1992) note that when using this approach, results for the group comparison are the same as regressing the log of the power function ratio *Y*/*X*^*k*^ on the grouping variable, granted *k* is estimated from a log-log model with the grouping variable included (see section titled “*Using the denominator as a covariate*”).

## Evaluating muscle size and strength with multiple regression

### Using the denominator as a covariate

Using ratios of variables to control for the effect of the denominator variable presents complexities. Accordingly, many have advocated for the use of multiple regression procedures to “control” or “account” for the denominator variable instead of forming a ratio (Packard and Boardman 1999; Curran-Everett 2013; Allison et al. 1995; Kronmal 1993). Moreover, some have highlighted that the use of regression modeling is first required to derive adjusted ratios, e.g., (*Y-a*)/*X* (Atchley 1978; Albrecht et al. 1993). Multiple regression models incorporating muscle size as a covariate could allow for comparing muscle strength between groups at a fixed value of muscle size, versus conducting an independent t test comparing the two groups on scores for the ratio (Table 1). In such a regression model (Eq. 4), muscle strength (the numerator) would be the dependent variable, and there would be two predictor variables: muscle size (the denominator) and a grouping variable:

4 $$\:Y=\:{b}_{0}+\:{b}_{1}X+\:{b}_{2}G$$

Where *Y* is strength, *X* is muscle size, and *G* is the grouping variable (e.g., trained versus untrained). The regression coefficient *b*_*1*_ is an estimate of the expected difference in strength for a 1-unit increase in size, after accounting for group membership, *G*. The regression coefficient *b*_*2*_ provides the estimated mean difference in muscle strength between the two groups after controlling for muscle size or holding it constant (assuming it is not manually entered into the model before *X*). This approach can be extended to having more than two groups via analysis of covariance (ANCOVA) procedures (Kleinbaum et al. 2014).

Advantages of multiple regression or ANCOVA approaches could include better control of the influence of *X*, adherence to the assumed error distribution of regression analyses, and increased statistical power over comparing groups on the ratio (Packard and Boardman 1999; Allison et al. 1995). There are, however, additional considerations and assumptions introduced when using these approaches. First, ANCOVA procedures may estimate different quantities than group comparisons on ratio scores (i.e., *Y* controlling for *X* versus *Y* relative to *X*). Each may provide conceptually distinct information, but ratio scores do not always provide statistical control (Smith 2005). Nevill et al. (1992) note that when investigating treatment effects, comparing groups on scores for *Y*/*X* may yield results that contradict with comparing groups on *Y* adjusted for *X* as a covariate. Thus, the approach taken should reflect the underlying research question. Moreover, the use of linear regression models to control for the effect of size carries its own assumptions (Table 1). For example, the relationship between the covariate and dependent variable is assumed to be the same between groups, i.e., that there is no group by covariate interaction in predicting the dependent variable (Table 1) (Kleinbaum et al. 2014).^7^ With these considerations in mind, multiple regression could also be used to estimate and account for allometric relationships between size and strength in group comparisons (Table 1). Examples from Nevill et al. (1992) and Batterham et al. (1997) using linear regression with log-transformed variables demonstrate group comparisons with control of allometric scaling relationships between covariates and dependent variables. In the current example, by taking the logarithm of *Y* and *X*, a model comparing groups could be:

5 $$\:\mathrm{l}\mathrm{o}\mathrm{g}\left(Y\right)=\:{b}_{0}+\:{b}_{1}\mathrm{l}\mathrm{o}\mathrm{g}\left(X\right)+\:{b}_{2}G$$

where *b*_1_ would provide an estimate of the allometric scaling exponent after accounting for group membership,^8^ and*b*_2_ would provide an estimate of proportional difference in strength between groups after accounting for the allometric effect of size. Interestingly, when using this approach, results for the group comparison are the same as regressing the log of the allometrically adjusted ratio, *Y*/*X*^*k*^ on *G*, granted *k* is estimated as in *b*_1_ from Eq. 5 (Batterham et al. 1997; Nevill et al. 1992).

In addition to group comparisons, multiple regression approaches also offer flexibility when evaluating relationships between muscle strength and continuous predictors of interest, i.e., as opposed to a grouping variable. In the case of ST or MQ, perhaps a goal may be to study the relationship between muscle function and age. By simultaneously regressing strength (only the numerator of ST or MQ) on both age and the muscle size variable (the denominator), the coefficient for the age variable would provide an estimate of the average difference in muscle strength for a 1-unit increase in age, after controlling for muscle size, or holding muscle size “constant” or “fixed” (Kleinbaum et al. 2014; Hayes 2022).

While in the above examples, regression coefficients are often the focus of research questions, there are also cases where the predicted values of such models controlling for size are of interest. Assuming the model adequately fits and describes the nature of the data, residuals can be used to derive individual “adjusted” or “corrected” scores for the dependent variable (Albrecht et al. 1993). For example, Atkinson and Batterham (2013) highlight the potential utility in deriving allometrically adjusted percentage flow-mediated dilation scores (a ratio involving change in artery diameter relative to baseline), a case where the ratio itself is of particular substantive interest. Lolli et al. (2017a) demonstrate this approach focusing on sex differences in ratios of second digit to fourth digit length, adjusted for the influence of the denominator. They highlight that through several possible models, estimates of either the ratio or numerator scores can be obtained after controlling for the influence of the denominator (Lolli et al. 2017a). This was advocated over calculation of allometrically adjusted ratios (see section titled “*Allometric scaling and ratios adjusted for scaling exponents”*), e.g., *Y*/*X*^*k*^, which may have size-related distributional effects (Lolli et al. 2017a).

## Other considerations for ratios in statistical analyses

### What about ratios of variables as predictors?

Thus far, we have focused on the ratio as a dependent variable. However, there may be interest in the ratio as a predictor of other outcomes. For example, one may have theoretical grounds for exploring whether strength relative to size is a valuable predictor of other outcomes, e.g., 6-minute walk distance. The goal may not necessarily be to hold muscle size “constant” or control for it. Rather, the interest revolves around the ratio of strength to size which is assumed to have meaningful implications (Smith 2005), as a predictor of other functional outcomes. Interestingly, if the ratio is used as a predictor in this manner, it could be argued that it represents something analogous to an *interaction term* (Morris et al. 2023) between two variables (e.g., muscle size and strength) equivalent to multiplying the numerator (i.e., muscle strength) by 1/denominator (i.e., muscle size) (Certo et al. 2020; Kronmal 1993; Allison et al. 1995).

With this in mind, some would suggest that lower-order terms making up the interactions should be included in the model as well (Hayes 2022; Kleinbaum et al. 2014; Kronmal 1993). Such models are referred to as hierarchically well-formulated (Kleinbaum et al. 2014) or models that follow the marginality principle (Morris et al. 2023). The argument is that without the inclusion of the lower-order terms, interpretation of regression coefficients for interactions or product terms could be unclear or that these parameter estimates for the interaction term could be biased (Hayes 2022) or could depend on how the variables making up the interaction are scaled or coded (Agresti 2007). Morris, van Smeden, & Pham (Morris et al. 2023) discuss some cases where it may make sense to ignore the marginality principle when using ratios. In following discussion by Kronmal (1993), an example of a hierarchically well-formulated model with a ratio of muscle strength (*Y*) divided by muscle size (*X*) predicting a dependent variable of 6-minute walk distance (*W*) could be:

6 $$\:W=\:{b}_{0}+\:{b}_{1}Y+\:{b}_{2}{X}^{-1}+\:{b}_{3}Y\cdot\:{X}^{-1}$$

Equation 6 shows that a variable 1/*X* would be included with *Y* and the ratio in the model. If the coefficient for the ratio, *b*_*3*_, were statistically significant, then there would be evidence of unique predictive ability of the ratio. The coefficient *b*_*3*_ would provide an estimate of how much the effect of 1/size on 6-minute walk distance varies for a 1-unit change in strength. Nevill et al. (1992) provide a similar example with an allometric model to predict running performance.

One consideration is whether using a product term for the interaction of size and strength i.e., *X* ⋅ *Y*, would be appropriate for evaluating whether size’s relationship with the dependent variable depends on differing values of strength. The two variables would be multiplied together, instead of divided, and entered into the regression model along with each individual lower-order term (Eq. 7). In this model, the interaction term would test whether the effect of one predictor on *X* depends on the values of the other predictor *Y* in the interaction. This could provide relevant quantification of whether, and to what degree, muscle size depends on strength in predicting health or performance related outcomes. An example application could be investigating whether effects of low muscle mass on *W* depend on how strong the muscles are. In this case, an example model could be:

7 $$\:W=\:{b}_{0}+\:{b}_{1}Y+\:{b}_{2}X+\:{b}_{3}Y\cdot\:X$$

In this model, *b*_*3*_ is an estimate of how much the relationship between size and 6-minute walk distance varies for a 1-unit difference in strength. In the case where *b*_*3*_ is statistically significant, we might probe the interaction (as is standard practice) to find the range of strength values at which the effects of size on 6-minute walk time are statistically significant/non-significant and perhaps practically meaningful (Hayes 2022). Conversely, if we were to find that *b*_*3*_ was not a significant predictor, then there would be insufficient evidence to conclude that the effect of muscle size on 6-minute walk distance depends on how strong the muscles are. For in-depth discussion on testing and interpreting interaction terms, see Hayes (2022) and Aiken and West (1991).

### Longitudinal analyses

The primary focus of this review has been on considerations for using ratios of ST or MQ to control for the effect of muscle size at a single time point. From this perspective, we have explored some of the underlying complexities in using ratios of variables, as well as some potential alternative strategies. However, as ST or MQ are often studied in a longitudinal context (e.g., in response to an intervention), it is worth highlighting that some of the issues discussed herein may become more complex, especially if the scaling pattern between strength and size changes substantially through training. Additionally, if the focus is on how muscle strength changes between randomized training and control groups with some consideration of its relationship with size, strategies may also depend on theoretical perspective of the role of size. Size (baseline or changes) might be considered a covariate, a mediator, or a moderator for the treatment effect. While thorough discussion of longitudinal analysis is beyond the scope of this review, the appropriate use of ANCOVA procedures (Kleinbaum et al. 2014; Senn 2006; Laird 1983; Curran-Everett and Williams 2015)^9^ and multilevel modelling techniques (Cohen et al. 2003; Singer and Willett 2003), possibly incorporating allometric scaling (Nevill et al. 1992; Armstrong and Welsman 2020), may be preferable alternatives to evaluating mean changes or differences in ratio scores.

## Conclusions and recommendations

Investigating ratios of strength relative to muscle size has shed light on the relationship between muscle size and strength amongst other aspects of neuromuscular physiology. When used to evaluate strength controlling for size, however, it has long been suggested that simple ratios rarely meet required conditions to adequately control for the effect of a denominator (Albrecht et al. 1993), consistent with the analysis demonstrations herein. Based on previous recommendations explored (non-exhaustively) in this review, we hope the following general suggestions may be useful for those interested in using ratios to control for muscle size when evaluating strength:

- Evaluate the form of the relationship between strength and size variables. This may depend on the measurements used.^1^
- Decide whether conditions are satisfied for the ratio to provide adequate control of the denominator without further adjustment of either numerator or denominator scores (e.g., scaling the denominator). Graphically and statistically evaluating systematic relationships between scores of the ratio over the range of denominator values would be helpful. The pattern and magnitude of the relationship, along with possible effects on comparisons should be considered.
- If the decision is to use a ratio (whether simple, adjusted for a y-intercept, or allometric scaling exponent), demonstrating its control of the denominator and meeting distributional assumptions would strengthen conclusions.
- Evaluate whether recommended regression-based methods that account for linear or allometric influence of size align with theoretical goals and consider these approaches in lieu of the ratio.
- Provide transparency to readers in the decision-making process for reproducibility.

The suggestions and alternatives discussed in this review are not intended to be strict guidelines as unique research questions and study designs frequently guide analytical choices. Rather, issues and considerations presented in this review are meant to help in identify and navigate analytical complexities when addressing research questions of interest.

## Data Availability

The datasets utilized in the review can be made available from the corresponding author upon reasonable request.

## Footnotes

1 There are several potential combinations of muscle strength and size (or mass) variables that can be used. Moreover, Smith (2005) notes that a ratio must have numerator and denominator variables with the same units, while a rate consists of numerator and denominator variables that have differing units. For consistency with existing literature, we use the term ratio throughout. For example, strength may be quantified through maximal voluntary isometric contraction force or dynamic one-repetition maximum, and muscle size or mass may be quantified through magnetic resonance imaging (MRI) or dual x-ray absorptiometry scanning (DEXA). For the purposes of this review, we also loosely refer to muscle strength and size and/or mass conceptually but acknowledge that many different variables could make up these ratios across the literature. Importantly, the statistical implications could differ based on the variables involved, which may have different dimensional scaling relationships (Smith 2005).

2 The weight of evidence across a large body or research suggests training with low loads (to or near task failure) can increase muscle size comparably to traditional high-load training (e.g., 3–4 sets of 70–80% one-repetition maximum or 8–12 repetition maximum), although increases in dynamic strength (e.g., one-repetition maximum) are greater with high-load training (Lopez et al. 2021; Currier et al. 2023; Schoenfeld et al. 2017).

3 This assumption may not always be the case. Relationships between physiological traits and size variables can exhibit curvilinear relationships (see section titled “*Allometric scaling an**d ratios adjusted for scaling exponents*”).

4 As with deriving a ratio (*Y-a*)/*X*, i.e., a ratio adjusted for the y-intercept (see section titled “*Ratios adjust**ed for the y-intercept*”, the estimate of *k* used in deriving a ratio *Y*/*X*^*k*^ should come from OLS versus model II regression procedures (Albrecht et al. 1993).

5 The sma() function of smatr() package (Warton et al. 2012) has a “slope test” argument for hypothesis testing of the slope coefficient against a specified null value (in this case 1).

6 Similarly with discussion of deviation from a zero intercept (see section titled “*Isometry testing: decision-making with hypothesis t**ests*”) a slope coefficient significantly different from 1 depends on several factors, and deciding whether a deviation is of sufficient magnitude may not always be straightforward.

7 These are coupled with other assumptions of linear regression including linearity in the parameters, homoscedasticity, normal distribution of the errors, and independence of errors (Kleinbaum et al. 2014).

8 Estimating a common allometric scaling exponent after accounting for group membership requires that there is no group by covariate interaction in predicting the dependent variable (Batterham et al. 1997; Nevill et al. 1992).

9 In the literature of ratios of variables in statistical analyses, discussion of percent changes [a ratio of (post-pre)/pre] in dependent variables also arises, given that variables of percent change are subject to some of the complexities discussed herein (Atkinson and Batterham 2012; Curran-Everett and Williams 2015).
